# Reproducibility and repeatability of ^18^F-(*2S*, *4R*)-4-fluoroglutamine PET imaging in preclinical oncology models

**DOI:** 10.1371/journal.pone.0313123

**Published:** 2025-01-09

**Authors:** Gregory D. Ayers, Allison S. Cohen, Seong-Woo Bae, Xiaoxia Wen, Alyssa Pollard, Shilpa Sharma, Trey Claus, Adria Payne, Ling Geng, Ping Zhao, Mohammed Noor Tantawy, Seth T. Gammon, H. Charles Manning

**Affiliations:** 1 Department of Biostatistics, Vanderbilt University Medical Center, Nashville, TN, United States of America; 2 Vanderbilt Ingram Cancer Center, Vanderbilt University Medical Center, Nashville, TN, United States of America; 3 Vanderbilt Center for Molecular Probes, Vanderbilt University Medical Center, Nashville, TN, United States of America; 4 Vanderbilt University Institute of Imaging Science, Vanderbilt University Medical Center, Nashville, TN, United States of America; 5 Department of Cancer Systems Imaging, The University of Texas MD Anderson Cancer Center, Houston, TX, United States of America; 6 Department of Radiology and Radiological Sciences, Vanderbilt University Medical Center, Medical Center North, Nashville, TN, United States of America; Brandeis University, UNITED STATES OF AMERICA

## Abstract

**Introduction:**

Measurement of repeatability and reproducibility (R&R) is necessary to realize the full potential of positron emission tomography (PET). Several studies have evaluated the reproducibility of PET using ^18^F-FDG, the most common PET tracer used in oncology, but similar studies using other PET tracers are scarce. Even fewer assess agreement and R&R with statistical methods designed explicitly for the task. ^18^F-(*2S*, *4R*)-4-fluoro-glutamine (^18^F-Gln) is a PET tracer designed for imaging glutamine uptake and metabolism. This study illustrates high reproducibility and repeatability with ^18^F-Gln for *in vivo* research.

**Methods:**

Twenty mice bearing colorectal cancer cell line xenografts were injected with ~9 MBq of ^18^F-Gln and imaged in an Inveon microPET. Three individuals analyzed the tumor uptake of ^18^F-Gln using the same set of images, the same image analysis software, and the same analysis method. Scans were randomly re-ordered for a second repeatability measurement 6 months later. Statistical analyses were performed using the methods of Bland and Altman (B&A), Gauge Reproducibility and Repeatability (Gauge R&R), and Lin’s Concordance Correlation Coefficient. A comprehensive equivalency test, designed to reject a null hypothesis of non-equivalence, was also conducted.

**Results:**

In a two-way random effects Gauge R&R model, variance among mice and their measurement variance were 0.5717 and 0.024. Reproducibility and repeatability accounted for 31% and 69% of the total measurement error, respectively. B&A repeatability coefficients for analysts 1, 2, and 3 were 0.16, 0.35, and 0.49. One-half B&A agreement limits between analysts 1 and 2, 1 and 3, and 2 and 3 were 0.27, 0.47, and 0.47, respectively. The mean square deviation and total deviation index were lowest for analysts 1 and 2, while coverage probabilities and coefficients of the individual agreement were highest. Finally, the definitive agreement inference hypothesis test for equivalency demonstrated that all three confidence intervals for the average difference of means from repeated measures lie within our *a priori* limits of equivalence (i.e. ± 0.5%ID/g).

**Conclusions:**

Our data indicate high individual analyst and laboratory-level reproducibility and repeatability. The assessment of R&R using the appropriate methods is critical and should be adopted by the broader imaging community.

## Introduction

Imaging is useful for lesion detection, staging, and evaluation of treatment response and disease progression. The sensitive and quantitative nature of PET, coupled with the ability to produce targeted PET tracers, renders PET uniquely capable of detecting tumors and profiling their specific features. Importantly, PET provides a functional measure of tumor phenotype non-invasively *in vivo*, which allows for a quantitative assay of biological processes, such as the activity of transporters and enzymes. However, to realize the full potential of PET imaging, imaging must demonstrate agreement, which is dependent upon the measurement of repeatability and reproducibility (R&R) [1, 2].

Glutaminolysis is vital to tumor growth, progression, and survival [3–13]. To meet their demand for glutamine, tumor cells transport glutamine into the cell from the tumor micro-environment [3–9, 11–14]. Glutamine can then be used in various downstream processes, including the synthesis of proteins, nucleic acids, and hexosamines, or conversion to glutamate, which can then be used as a source of glutathione, α-ketoglutarate, or nonessential amino acids [3–11]. To study the uptake and metabolism of glutamine, syntheses of ^18^F-labeled glutamine analogues for PET imaging have been reported [15–18]. ^18^F-Gln has been studied preclinically [19–30] and clinically, including in brain, pancreas, breast, lung, and thyroid cancers [21, 26, 31–34]. Recent studies evaluated the reproducibility of ^18^F-FDG PET imaging in phantoms [35] and preclinical models [36–38]. Similar studies using other PET tracers are scarce, and even fewer assess agreement and R&R with statistical methods designed explicitly for the task. These studies are needed for new tracers to support basic science research and, more importantly, are necessary for the clinical translation of these tracers and, ultimately, adoption of these methods as part of standard-of-care imaging. Here, we assess ^18^F-Gln using data from an experiment explicitly designed to evaluate user agreement, reproducibility, and repeatability.

## Materials and methods

### Cell lines

HCT-116 cell lines were purchased from ATCC (American Type Culture Collection) and authenticated using a commercial vendor (Genetica). Cells were cultured in Dulbecco’s Modified Eagle Medium (DMEM) containing 10% fetal bovine serum (FBS) and 1% penicillin/streptomycin (p/s). The cells were incubated in 5% CO_2_ at 37°C.

### Animal models

All animal procedures complied with the Guide for the Care and Use of Laboratory Animal Resources (1996) and National Research Council and were approved by the Vanderbilt University Institutional Animal Care and Use Committee (Nashville, TN, USA). Animals were purchased from Envigo and used in accordance with Institutional and Federal guidelines. Female athymic nude mice (Hsd: Athymic Nude-*Foxn1*^*nu*^, Envigo, #6903), 5-6-weeks old, were injected subcutaneously into the right flank with 8 x 10^6^ HCT-116 cells. Twenty mice were used in this study. Mice were monitored daily and tumor size and body weight were measured three times per week. The tumor volume was calculated according to the formula WxLxH/2. Imaging was performed when the tumor volume reached ~250 mm^3^ at days 19–22 post-tumor cell injection. Animals were anesthetized with 2% isofluorane prior to tracer administration and were kept warm using in their cages using circulating water bath until imaging. All efforts were made to minimize suffering, mice were kept warm during PET imaging via circulated heated water. None of the mice reached humane endpoints (tumor size greater than 1.5 cm in average diameter, body weight loss more than 20%, or state of moribundity) before completion of the experiment. At the end of the experiment, mice anesthetized by isoflurane gas were euthanized by carbon dioxide asphyxiation followed by cervical dislocation. Prior to asphyxiation and subsequent cervical dislocation, mice were palpated to ensure deep anesthesia and prevent suffering.

### Radiochemistry

[^18^F]-(*2S*, *4R*)-4-fluoro-glutamine was synthesized as previously described by our group using methodologies identical to those reported [15, 22].

### PET imaging

PET imaging experiments were performed using HCT-116 tumor-bearing mice. Two sets of 10 mice each were imaged on consecutive days. Imaging conditions were kept as consistent as possible between days. Access to food and water was provided ad libitum. Animals were anesthetized with 2% isofluorane and administered 8.2–11.4 MBq of ^18^F-Gln via retroorbital injection by highly trained personnel and as approved by the Vanderbilt University Institutional Animal Care and Use Committee (Protocol Numbers M1800041 and M1500003). The mice were returned to their cages and kept warm via a circulating water bath. Following 40 minutes of tracer uptake, mice were anesthetized with 2% isoflurane and static images were acquired for 20 minutes in an Inveon microPET (Siemens Preclinical Solutions). One mouse was accidentally imaged using the wrong imaging scanner protocol initially. This mouse was reimaged using the correct imaging scanner protocol (20-minute static images) at a later time point. Thus, the PET images for this mouse were acquired at a radiotracer uptake time of more than 2.5 hours post-injection instead of 40 minutes post-injection. The images for this mouse using the correct imaging scanner protocol at the later time point were included in the image analysis and statistical comparisons.

### Image analysis

All data sets were reconstructed using the three-dimensional (3D) ordered subset expectation maximization/maximum a posteriori (OSEM3D/MAP) algorithm into 128 x 128 x 95 slices with a voxel size of 0.095 x 0.095 x 0.08 cm^3^ at a beta value of 0.01. The PET images were loaded onto the image analysis tool Amide (www.souceforge.net) to export the Digital Imaging and Communications in Medicine (DICOM) format with the percentage of the injected dose per gram of tissue (%ID/g) unit. PMOD software (PMOD Technologies LLC, Zurich, Switzerland) was used to draw 3D volumes of interest (VOIs) around the tumors on the right flank and around the muscle on the contralateral left flank in the PET images. VOIs were captured by using the intrinsic 3D algorithm of the auto iso-contour tool based on spheres (analytic objects) of a certain size on the software. The measured counts were converted to the percentage of the injected dose per gram of tissue (%ID/g). Tumor/muscle ratio was calculated as the tumor %ID/g value divided by the muscle %ID/g value.

**Fig 1:**
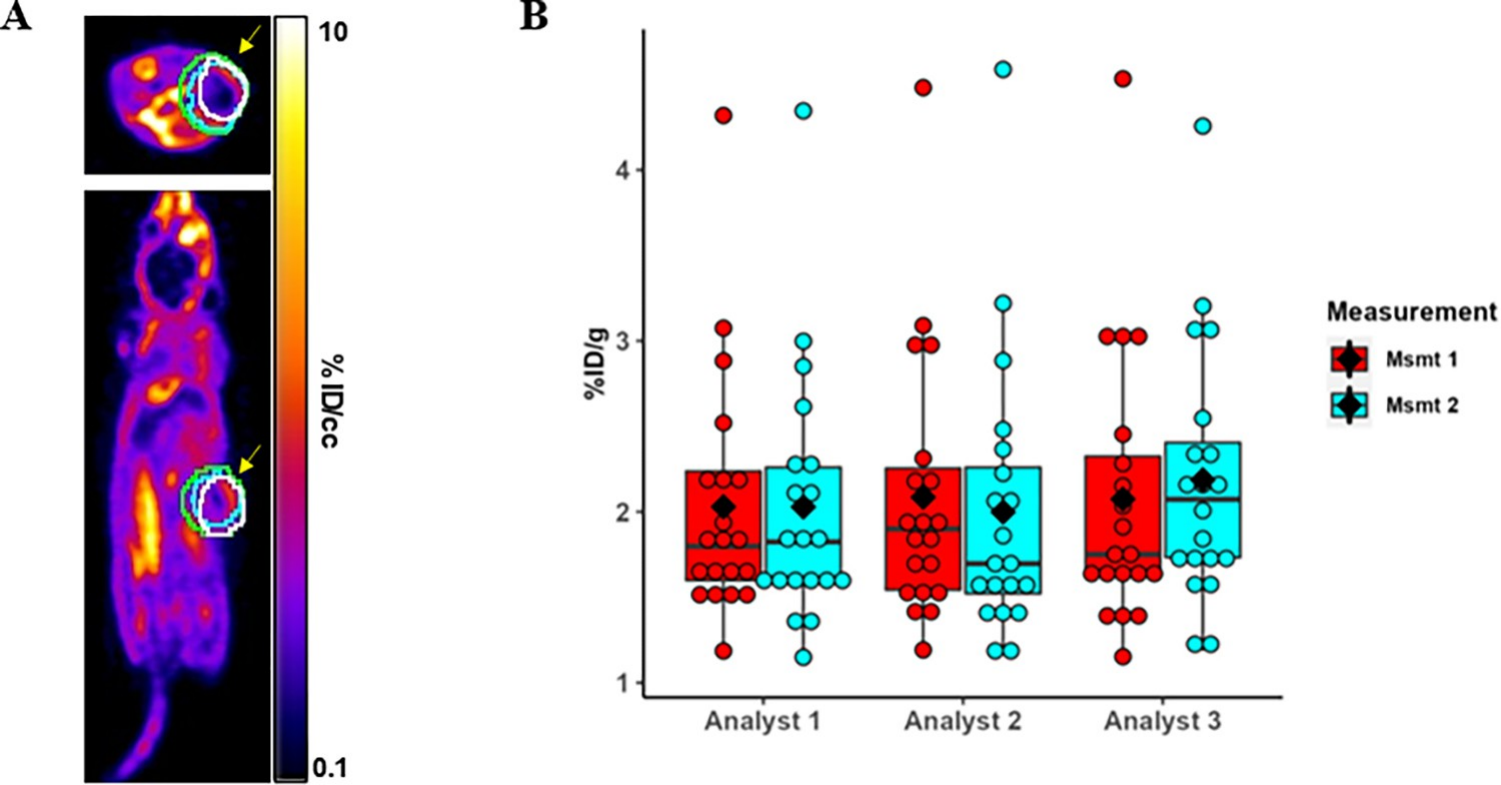
**A)** Representative axial and coronal images of %ID/g using [^18^F]-(*2S*, *4R*)-4-fluoro-glutamine. Yellow arrows indicate tumor location. White (analyst 1), cyan (analyst 2), and green (analyst 3) volumes of interest (VOIs) on the tumor were captured by the 3D algorithm of auto iso-contour that each analyst drew. **B)** Dot/boxplot of %ID/g for twice replicated data of 20 mice among 3 analysts. Diamonds represent the mean value and horizontal bars the median. Bottom and tops of boxes represent the 25^th^ and 75^th^ percentiles. Vertical lines extending above and below the boxes are 1.5*the interquartile (IQR = 75^th^– 25^th^ percentile) range. The data points that are greater than 4%ID/g are all from the same mouse. This mouse was imaged using a different protocol however despite this variation in protocol, repeat measurements by the three analysts result in similar uptake values.

Three people analyzed the OSEM3D/MAP datasets from the HCT-116 test-retest study using the same image analysis software (PMOD) to evaluate the effect of user R&R on imaging results (Fig 1). Scans were randomly re-ordered for a second, repeatability measurement six months later. One person has more than 5 years of experience in analyzing preclinical PET data. The other two people have less than five years of experience each.

### Statistical methods

Based on our reading, the methods of Bland and Altman, the intra class correlation coefficient (ICC), and Lin’s concordance correlation coefficient (CCC) are the most cited methods for agreement. Gauge reproducibility and repeatability (Gauge R&R) and other useful methods based on mixed and random effect models are rare in the imaging literature. A definitive test for equivalency could not be found in the imaging literature. We provide, for the first time to our knowledge, direct comparison of these various metrics for imagers.

Bland and Altman (B&A) limits of agreement (LOA) for reproducibility and repeatability coefficients (RC) were estimated and graphically presented based on their landmark methods for estimands of R&R in medical applications [39–41] for average (over repeated measurements) and single measurements among reviewers. LOA is defined as LOA = $\bar{d}\pm 1.96*S_{d}$ where $\bar{d}$ and *S_d_* are the average difference and standard deviation of the differences between reviewers. Repeatability coefficient (RC) is calculated according to the equation B&A RC = 1.96*$\sqrt{2}$*S_w_ where S_w_ is the within-subject standard deviation of the replicates from the same reviewer. The CCC has reached the imaging literature with CCC greater than 0.8 or 0.9 considered excellent [42–48]. Consequently, statistical tests comparing the CCC to zero concordance seem unwarranted (S1 File).

### Gauge Reproducibility and Repeatability (Gauge R&R)

We also used Gauge Reproducibility and Repeatability (Gauge R&R) methods to estimate the capability of our measurement system as a whole for ^18^F-Gln PET imaging [49, 50]. Using the 2-way crossed random effects model: Y_ijk_ = μ + M_i_ + O_j_ + (MO)_ij_ + E_ijk_, where i = 1, … 20 mice, j = 1, …,3 analysts, and k = 1, …,2 repeated measures we estimated $\sigma_{M}^{2},\;\sigma_{O}^{2},\;\sigma_{MO}^{2}$, and $\sigma_{E}^{2}$, respectively and define Repeatability = $\sigma_{E}^{2}$, reproducibility = $\sigma_{O}^{2}+\sigma_{MO}^{2}$, and the total variability of the measurement procedure = $\sigma_{O}^{2}+\sigma_{MO}^{2}+\sigma_{E}^{2}$. From these estimates arise several useful parameters [49] which can be extracted from the mean square estimates of random and mixed models in the EMSaov package or lme4 package found in the R software system [51–55]. See supplementary materials for more detail. A definitive comparison of agreement between reviewers was made using a test of equivalence. Novel to this paper, $\bar{d}\pm t_{df=N-1,\alpha =0.05/2}*S_{d}/\sqrt{N}$, is the 95% confidence interval for the average mean difference between two observers. A 95% confidence interval that lies within predetermined ±δ is equivalent to rejecting the null hypothesis (p<0.05) of non-equivalence, *H*_0_: *μ* ≤ -δ or *μ* ≥ δ and declaring equivalence between observers. With 3 reviewers, we estimated a set of 3, 98.3% (1–0.05/3) confidence intervals, to control the experiment-wise error rate using a Bonferroni correction at 5% (S1 File).

## Results

### Summary of imaging measures

Tumor PET imaging data from 20 mice were analyzed by three different people at our institution, with replicate observations performed 6 months later, using the same set of images (OSEM3D/MAP reconstructed data), the same software (PMOD), and the same analysis method. Results are summarized in Table 1 (%ID/g) and S1 Table (tumor/muscle ratio) and depicted in Fig 1. Overall, mixed model-based estimates (95% CI) which incorporate repeated measures correlation, were 2.07 (1.71 to 2.42) and 1.13 (1.03 to 1.23), respectively, for %ID/g and tumor/muscle ratios. Raw data based average (SD) %ID/g ranged from 2.00 to 2.19 across all analysts and measurements, while the standard deviation ranged from 0.72 to 0.82. Coefficients of variation (CV) ranged from 0.34 to 0.41. Analyst 1 had the most consistent average and median values, the lowest standard deviations, and the lowest CV across their respective measurements. Fig 1B is a dot/boxplot that depicts the data for %ID/g.

**Table 1:** Summary statistics of %ID/g by analyst and measurement.

Statistic	Analyst 1		Analyst 2		Analyst 3	
	Msmt 1*	Msmt 2	Msmt 1	Msmt 2	Msmt 1	Msmt 2
**Mean (SD)**	2.03 (0.72)	2.03 (0.74)	2.09 (0.78)	2.00 (0.82)	2.07 (0.81)	2.19 (0.75)
**Median (range)**	1.80 (1.19, 4.32)	1.82 (1.15, 4.35)	1.90 (1.19, 4.48)	1.70 (1.17, 4.59)	1.75 (1.15, 4.53)	2.07 (1.19, 4.26)

*Msmt = measurement

### Estimation of PET imaging system capability via Gauge R&R

Generally speaking, repeatability is a characteristic of dependent measurements by the same analyst, for the same tissue, or for the same mice. Reproducibility is a characteristic of independent entities as with analysts, operators, and devices.

Overall system reproducibility and repeatability metrics assuming a two-way random effects Gauge R&R model are presented in Table 2 (S2 Table for first measurement data). Variability among mouse tumors (mouse variance; ($\sigma_{M}^{2}$)) was 0.5717, analyst variance ($\sigma_{O}^{2}$) was 0.0024, process by imaging interaction ($\sigma_{MO}^{2}$) was 0.0052, and repeatability ($\sigma_{E}^{2}$) was 0.0166 for a total measurement error ($\sigma_{O}^{2}+\sigma_{MO}^{2}+\sigma_{E}^{2}$) of 0.0242. The animal variance was 24 times greater than measurement variation, indicating that biological variability is the main source of differences in PET uptake values. Reproducibility ($\sigma_{O}^{2}+\sigma_{MO}^{2}$) and repeatability accounted for 31% and 69% of the total measurement error, respectively. The intra-class correlation coefficient (ICC) is the proportion of total variation due to mice and represents the correlation between two measurements taken on the same mouse; the ICC in this study was 0.96. Confidence intervals are the 2.5 and 97.5 percentiles from bootstrap sampling based on 10,000 replicates.

**Table 2:** Results of Gauge reproducibility and repeatability on repeated measures data set.

Model Parameter	Analyst as a Fixed Effect	Analyst as a Random Effect
Mouse Variance	0.5716694	0.5716694
Analyst Variance	0.0015801	0.0023701
Interaction Variance	0.0052416	0.0052416
Error Variance	0.0166434	0.0166434
Mouse Variance	0.572 (0.143, 1.108)	0.572 (0.147, 1.114)
Measurement Variance	0.023 (0.013, 0.036)	0.024 (0.014, 0.037)
Mouse to Measurement Ratio	24.363 (5.530, 60.416)	23.569 (5.607, 58.479)
Repeatability Proportion	0.709 (0.493, 1)	0.686 (0.491, 0.969)
Reproducibility Proportion	0.291 (0, 0.507)	0.314 (0.031, 0.509)
Intraclass Correlation Coefficient	0.961 (0.847, 0.984)	0.959 (0.849, 0.983)

### Comparing analysts

**Fig 2:**
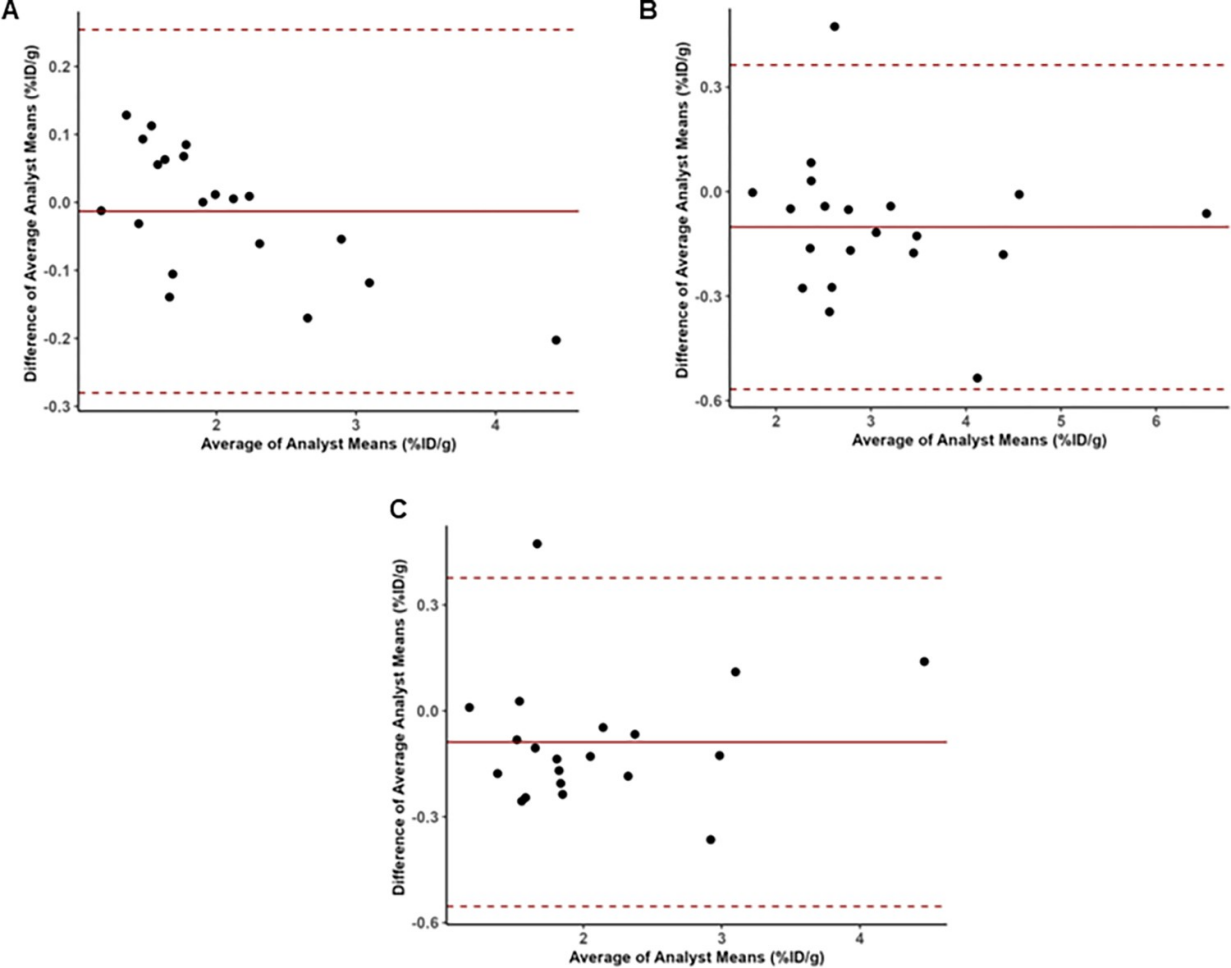
Bland-Altman plot comparing **A)** analysts 1 and 2, **B)** analysts 1 and 3, and **C)** analysts 2 and 3 from repeated measures (2 replicates) design.

The repeatability coefficients for analysts 1, 2, and 3 were 0.16, 0.35, and 0.49 (Table 3). Next, we compared data obtained between two analysts (Table 4 and S3 Table). One-half B&A LOA between analysts 1 and 2, 1 and 3, and 2 and 3 were 0.27%ID/g, 0.47%ID/g, and 0.47%ID/g, respectively. Bias between analyst pairs, were -0.013%ID/g, -0.101%ID/g, and -0.088%ID/g, respectively. B&A plots depict the ranges where 95% of differences between analysts will lie (Fig 2). Table 5 shows the agreement metrics based on a two-way mixed model with analysts as a fixed effect. The mean square deviation (MSD) and total deviation index (TDI) were lowest for analysts 1 and 2, while coverage probability (CP) and coefficient of individual agreement (CIA) were higher. The CIA was markedly different, reflecting the combined differences in S_w_ and bias between analysts. Analysts 1 and 2 exhibited the highest CIA, with lower average S_w_ and bias. Along with the ICC, analysts 1 and 3 had the worst CIA followed by analysts 2 and 3. Interestingly, analysts 2 and 3 had less bias and more narrow agreement limits on their first measurement than analyst 1 versus the other two analysts (S1–S3 Figs, S3 Table). The lower limits of the 95% confidence intervals for the CCC exceed 0.9 (S4 Table).

**Table 3:** Bland-Altman repeatability index (RI–per analyst).

Analyst	Repeatability Index*
1	0.155
2	0.346
3	0.490

*RI = 1.96* $\sqrt{2}$*S_w_, where S_w_ is the within-mouse (subject) standard deviation from repeated measures within analyst.

**Table 4:** Bland-Altman Limits of Agreement (LOA—repeated observations model).

Analysts	1/2 Agreement Limit*	Equivalency Lower Confidence Limit**	Bias***	Equivalency Upper Confidence Limit**
1 vs 2	0.267	-0.078	-0.013	0.051
1 vs 3	0.465	-0.213	-0.101	0.01
2 vs 3	0.465	-0.213	-0.088	0.01

*LOA = 1.96*S_d_, where S_d_ is the standard deviation of paired differences adjusted for repeated measures. **95% confidence intervals using t value with 19 degrees of freedom. ***Average difference of paired measurements.

**Table 5:** Agreement metrics based on pairwise analyst mixed models*.

Metric	1 vs 2	1 vs 3	2 vs 3
1/2 Agreement Limits	0.267	0.465	0.465
Intraclass Correlation Coefficient	0.984 (0.934, 0.992)	0.934 (0.757, 0.974)	0.943 (0.782, 0.976)
Mean Square Deviation	0.019 (0.1, 0.32)	0.067 (0.035, 0.107)	0.064 (0.039, 0.096)
Total Deviation Index**	0.269 (0.193, 0.351)	0.506 (0.369, 0.640)	0.497 (0.388, 0.608)
Coverage Probability***	0.999 (0.955, 0.999)	0.947 (0.874, 0.992)	0.952 (0.893, 0.988)
Coefficient of Individual Agreement**	0.991 (0.597,0.999)	0.516 (0.282, 0.830)	0.729 (0.484, 0.883)

*Two-way mixed model (analyst as fixed effect). **Using mean square deviation with *p* = 0.95. ***Tolerance limits ± 0.5%ID/g. 95% bootstrap confidence intervals in parentheses from 10,000 replications.

**Fig 3:**
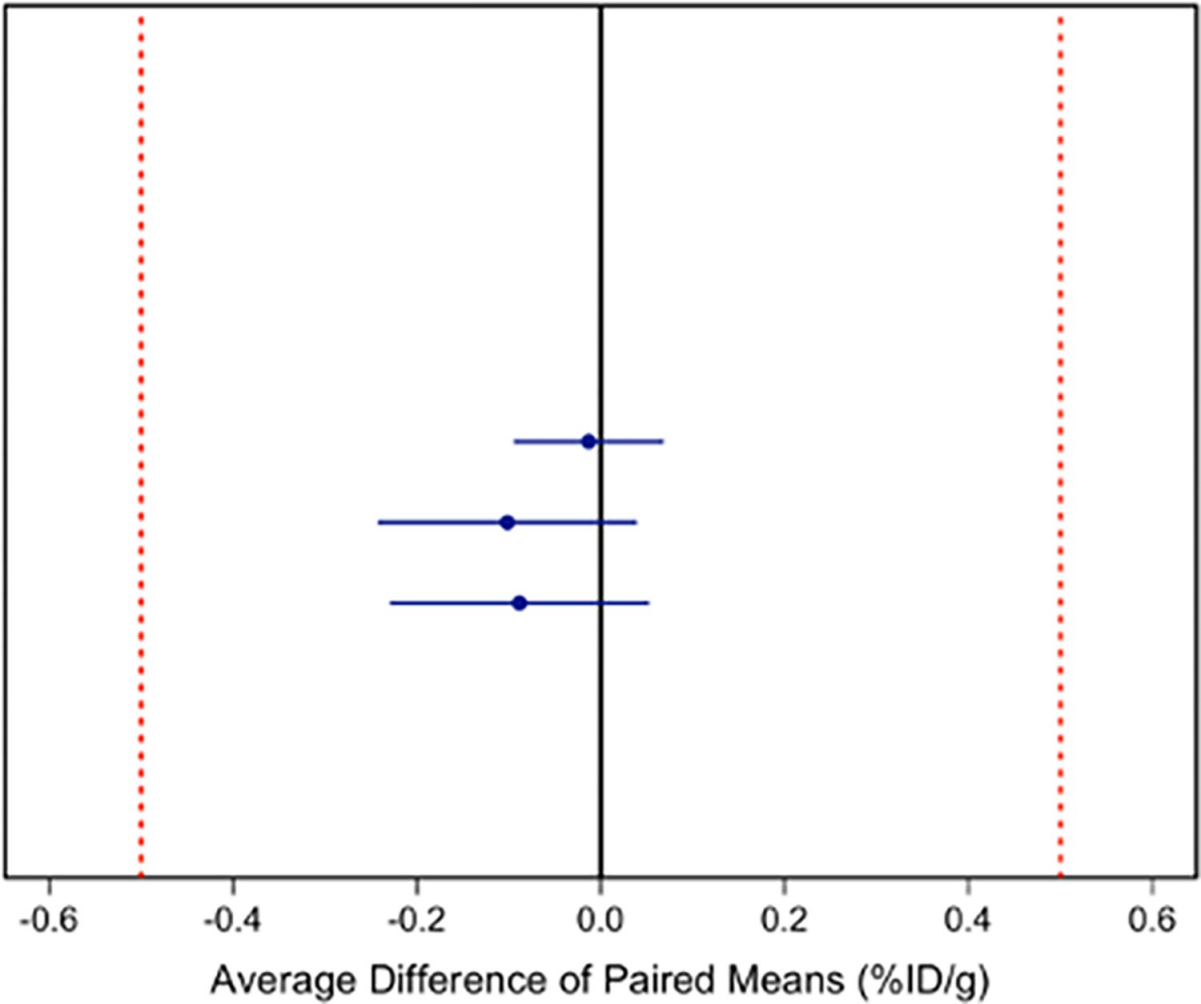
Hypothesis test to reject non-equivalency using confidence intervals for average difference of means from repeated measures (2 replicates) design. Shown are Bonferroni adjusted 98.3% (1–0.05/3) confidence intervals to control the experiment-wise type I error rate at 5%.

We tested the null hypothesis of non-equivalence with an experiment-wise error rate of 5% based on the set of Bonferroni corrected confidence intervals for the mean difference among analyst pairs (Fig 3, S4 Fig and S4 Table). All three confidence limits lie within our *a priori* limits of equivalence (i.e., ± 0.5%ID/g).

## Discussion

Limited R&R have been assessed by other labs for PET and MRI imaging. Savaikar et al. reported B&A limits of agreement for SUVmax (0.44) in ^18^F-FDG-PET was approximately 3-fold higher than that for SUVmean (0.15), suggesting poor reproducibility for SUVmax, confirming the well-known phenomenon that variability among extreme (maximum, minimum) values is greater than average values [38]. Whisenant et. al. used B&A methods to estimate repeatability of several imaging metrics in a murine model of HER2+ breast cancer [56, 57]. Limits of agreement were presented but no discussion of the adequacy of these limits or direct comparisons to other studies were made, likely due to the sparsity of such information in the literature.

We question a common hypothesis testing scenario for agreement. The first is testing for reproducibility using a null hypothesis of no difference between analysts, or devices, with the intent of declaring reproducibility following a p > 0.05 in comparative or pre-post comparisons. Such a comparison does not test for agreement. We cannot conclude, after failing to reject a null hypothesis, that the null hypothesis is true. We tested a non-equivalence null hypothesis for the average difference between analysts. We rejected the null hypothesis that bias (the average difference) in %ID/g among our analysts are greater than |0.5% ID/g| and conclude our analysts can be used interchangeably for the measurement of ^18^F-Gln. Another weak approach for testing agreement measures (e.g., CCC, Kappa, ICC) is to test a null hypothesis of zero agreement. Statistically significant but unimportant agreement can be concluded simply by using a sufficiently large sample size alone if desired. The research community ultimately determines minimum values of agreement and tolerance. A meaningful statistical test for an agreement metric could reject a null hypothesis of |0.8| or lower, for example, among agreement metrics ranging from -1 to 1 or 0 to 1, respectively. For %ID/g in the first measurement analysis, the lower 95% CI for the CCC exceeded 0.9. For this reason, assessment of the adequacy of estimates of agreement metrics should be accompanied by confidence limits.

Pairwise comparisons of analysts, devices, and other factors implies the fixed effect setting since the interest lies in differences between specific devices or readers. Broadening inferences to exchangeability of devices, analysts, or reconstruction methods for an entire lab or across labs changes the indication to the use of the random effects model for estimation. Lab quality improvement benefits from pairwise comparisons. Analyst 1 elicited a markedly lower repeatability coefficient, narrower agreement limits, mean square deviation, and TDI with higher ICC, CP, and CIA. Technique improvement training following this highly reproducible user’s methodology could be a source of training for current and future analysts in a lab.

Poor repeatability limits reproducibility between analysts and by extension, a lab’s reliance on exchangeability when reporting experimental results in the literature. Measurement variability without replication per Gauge R&R was 0.013 (S2 Table) compared to 0.024 for the same analysis on replicated data (Table 2). Corresponding agreement limits for differences were lower for 2 of the 3 analysts accompanied by a different ordering of reproducibility: analysts 1, 2, 3 vs 3, 1, and 2, respectively. Clearly, an accurate assessment of reproducibility requires an assessment of repeatability.

### Conclusion

While the panel of agreement metrics available is now extensive and we have added non-equivalence testing and Gauge R&R metrics to the evaluation set, the adoption of reproducibility and repeatability assessment in the imaging literature remains low. A subset of these metrics and methods may be sufficient by assessment of their intent and characteristics [55]. We suggest that correlation-based measures and statistical tests against null hypotheses of zero difference have lower utility. As agreement assessment suffuses the imaging research community, limits of agreement, equivalence confidence intervals, and confidence intervals of agreement statistics provide critical descriptive measures for improving reproducibility of experimental results for basic and clinical imaging. Research presented that cannot demonstrate adequate laboratory reproducibility and repeatability may be considered insufficiently rigorous. We defined tolerance limits for *in vivo* imaging of ^18^F-Gln, based on experience prior to analysis, as ± 0.5% ID/g. Multiplicity-adjusted difference confidence intervals were well within tolerance limits. We conclude that we have high individual analyst and laboratory-level reproducibility and repeatability. Expanding the inference to the population of investigators in the research community at large was adjusted for by larger variability in the mixed models that treated investigators as random effects. Per usual, precision of such estimates improves to a limit with greater sample size. Such inference, however, would logically and statistically improve in an investigation of randomly sampled investigators across labs and institutions. Given the apparent plethora of metrics available, an equivalence test, reproducibility (e.g., biological variance to measurement variance ratio—ICC), and repeatability (e.g. ICC and coefficients of individual agreement), with 95% confidence intervals and supporting graphics, should be considered.

## Supporting information

S1 File Statistical methods for reproducibility and repeatability. (DOCX)

S1 Fig Bland-Altman plot comparing analysts 1 and 2 from first measurement data. (TIF)

S2 Fig Bland-Altman plot comparing analysts 1 and 3 from first measurement data. (TIF)

S3 Fig Bland-Altman plot comparing analysts 2 and 3 from first measurement data. (TIF)

S4 Fig Hypothesis test to reject non-equivalency using confidence intervals for mean difference from repeated measures (2 replicates) design. Shown are Bonferroni adjusted 98.3% (1–0.05/3) confidence intervals to control the experiment-wise type I error rate at 5%.
(TIF)

S1 Table Summary statistics of tumor/muscle ratio by analyst and measurement. (DOCX)

S2 Table Results of Gauge reproducibility and repeatability on first measurement data set. (DOCX)

S3 Table Bland-Altman Limits of Agreement (LOA) for first measurement. (DOCX)

S4 Table Concordance correlation coefficient (95% confidence interval) for first measurement between analysts. (DOCX)

S1 Graphical abstract (TIF)
