# Supplementary material for: Reproducibility and repeatability of 18F-(2S, 4R)-4-fluoroglutamine PET imaging in preclinical oncology models
Source: PLoS One. 2025 Jan 9;20(1):e0313123. doi: 10.1371/journal.pone.0313123 (PMC11717184; doi:10.1371/journal.pone.0313123)
Supplement: S1 File — (DOCX) [file pone.0313123.s001.docx]

**Statistical Methods for Reproducibility and Repeatability**

Limits of Agreement

Bland and Altman (B&A) presented their landmark methods for estimands of R&R in medical applications [1] while illustrating the Pearson correlation coefficient and slope of the least squares regression line are not measures of agreement but of association. Both correlation and regression slopes are less than one, depending on the amount of measurement error of the method chosen as an independent. The method of B&A provides estimates of both bias (i.e., average difference between methods) and variability. The B&A repeatability coefficient (RC) requires replicate values on the same subject with the same method. It is derived from the within-subject standard deviation of the replicates (S_w_), (B&A RC = 1.96*$\sqrt{2}$*S_w_) assuming no bias between measurements, no trend in the differences, and differences are normally distributed. S_w_, on the scale of differences, estimates the range within which 95% of replicate differences are expected to lie. B&A use similar considerations to estimate the limits of agreement (LOA = $\bar{d}\pm1.96*S_{d}$, where $\bar{d}$and $S_{d}$ are the average difference and standard deviation of the differences between methods of measurement). Special cases include derivations by analysis of variance (ANOVA), adjusting for trends in average differences, estimation in replication within methods, and estimates of the precision of the limits of agreement, among others [2-5].

Lin’s Concordance Correlation Coefficient

Lin describes the Concordance Correlation Coefficient (CCC) as a reproducibility index, measuring the level of agreement between paired (same experimental unit) readings [6]. The CCC=ρ_c_ =C_b_*ρ where C_b_ is a bias correction factor that measures how far the best fit line deviates from the 45^o^ line of perfect agreement and ρ is the Pearson correlation statistic estimating precision of the points around this line. C_b_ is strictly greater than zero; no deviation from 1 is in perfect agreement with the 45^o^ line (perfect accuracy). The CCC lies between -1 and 1 inclusive and equals zero if and only if ρ = 0. Extensions to >2 observers and replicates on *n* subjects from *k* observers over *p* time points are available [7-9]. The CCC has reached the imaging literature with CCC greater than 0.8 or 0.9 considered excellent [10-12]. Consequently, statistical tests comparing the CCC to zero concordance seem unwarranted. The CCC shares issues with other correlation measures; it is dependent on the range of the measurement with no scales related to the measurements and does not provide estimation of the size of error which might be clinically allowable [3].

Gauge Reproducibility and Repeatability (Gauge R&R)

We also used a Gauge R&R study to estimate the capability of our measurement system as a whole for ^18^F-Gln PET imaging [13,14]. Three operators analyzed tumor images twice each from 20 mice imaged with ^18^F-Gln (Fig 1). We based our study on the 2-way crossed random effects model: Y_ijk_ = μ + M_i_ + O_j_ + (MO)_ij_ + E_ijk_, where i=1, … 20 mice, j=1, …,3 analysts, and k=1, …,2 repeated measures. M_i_, O_j_, (MO)_ij_, and E_ijk_ are jointly independent normal random variables with means of zero and variances $\sigma_{M}^{2}$, $\sigma_{O}^{2}$, $\sigma_{MO}^{2}$, and $\sigma_{E}^{2}$, respectively. Mice (subjects) are exclusively considered random. In our view, pairwise comparison of analysts requires their treatment as fixed factors. When considering the measurement system as a whole, analysts and other factors may be considered random effects as our process measurement inference is to the imaging laboratory as a whole. Repeatability = $\sigma_{E}^{2}$, reproducibility= $\sigma_{O}^{2}$ + $\sigma_{MO}^{2}$, and the total variability of the measurement procedure= $\sigma_{O}^{2}$ + $\sigma_{MO}^{2}$ + $\sigma_{E}^{2}$. From these estimates arise several useful parameters. For more information see Burdick et al. who illustrate derivations of these measurements using analysis of variance (ANOVA) methods [13] which can be extracted from the mean square estimates of random and mixed models in the EMSaov package or lme4 package found in the R software system [15,16].

Parker et al. provides an excellent tutorial for the variety of agreement measurements cited above [17]. Along with B&A limits of agreement (LOA), useful indexes based on the mean square deviation (MSD) are presented along with data and code for implementation [17-19]. The MSD is defined as the expected squared difference between two readers. For our linear model of this replicated experiment, MSD=${(\beta_{1}-\beta_{2})}^{2}+2\left( \sigma_{MO}^{2}+\sigma_{E}^{2} \right).$For a given range of clinical or biological importance, ±δ, the LOA may not lie within ±δ, which is centered on zero [17]. As bias approaches zero, the MSD approaches S_d_. From the MSD, we can calculate the total deviation index (TDI), coverage probability (CP), and coefficient of individual agreement (CIA) [18,19]. The TDI ( $\phi^{-1}(\left( \frac{1+p}{2} \right)\sqrt{MSD\left( Y_{1},Y_{2} \right)})$ ) provides the boundaries within which differences will be contained *p%* of the time. The coverage probability (CP(δ)=$1-2\{1-\phi\left( \frac{\delta}{\sqrt{MSD\left( Y_{1},Y_{2} \right)}} \right)\}$) is the probability that between observer (e.g. analyst) differences lie within tolerance limits. Here, δ are tolerance limits, and $\phi$(•) is the standard normal cumulative distribution function. Finally, the coefficient of individual agreement (CIA=$(\frac{2\sigma_{E}^{2}}{MSD})$) is the ratio of the residual error variance and the MSD. The residual variance, $\sigma_{E}^{2}$, represents the variation of repeated measurements taken under the same conditions (same analyst). As a ratio of this variation to the MSD (expected squared difference in readings by two individuals on the same animal), the CIA of 1 indicates that using different analysts makes no difference to the variability of repeated measurements within the same subject. A larger MSD in the denominator drives the ratio to zero, indicating much larger between than within analyst variability.

Test of Equivalence Using 95% Confidence Intervals

Let $\bar{d}\pm t_{df=N-1, \alpha=0.05/2}*S_{d}/\sqrt{N}$ be the 95% confidence interval for the average mean difference between two observers. A 95% confidence interval that lies within predetermined ±δ is equivalent to rejecting the null hypothesis (p<0.05) of non-equivalence, 𝐻_0_: 𝜇 ≤ -δ or 𝜇 ≥ δ and declaring equivalence between observers. It is appropriate to control the experiment-wise error rate using a Bonferroni correction for testing and confidence intervals.**References**

**1.** Altman DG, Bland JM. Measurement in Medicine - the Analysis of Method Comparison Studies. *Journal of the Royal Statistical Society Series D-the Statistician.* 1983;32:307-317.

**2.** Bland JM, Altman DG. Statistical Methods for Assessing Agreement between Two Methods of Clinical Measurement. *Lancet.* 1986;1:307-310.

**3.** Bland JM, Altman DG. A note on the use of the intraclass correlation coefficient in the evaluation of agreement between two methods of measurement. *Comput Biol Med.* 1990;20:337-340.

**4.** Bland JM, Altman DG. Measuring agreement in method comparison studies. *Stat Methods Med Res.* 1999;8:135-160.

**5.** Bland JM, Altman DG. Agreement between methods of measurement with multiple observations per individual. *J Biopharm Stat.* 2007;17:571-582.

**6.** Lin LI. A concordance correlation coefficient to evaluate reproducibility. *Biometrics.* 1989;45:255-268.

**7.** Carrasco JL, King TS, Chinchilli VM. The concordance correlation coefficient for repeated measures estimated by variance components. *J Biopharm Stat.* 2009;19:90-105.

**8.** King TS, Chinchilli VM, Carrasco JL. A repeated measures concordance correlation coefficient. *Stat Med.* 2007;26:3095-3113.

**9.** Carrasco JL, Phillips BR, Puig-Martinez J, King TS, Chinchilli VM. Estimation of the concordance correlation coefficient for repeated measures using SAS and R. *Computer Methods and Programs in Biomedicine.* 2013;109:293-304.

**10.** Baessler B, Weiss K, Pinto Dos Santos D. Robustness and Reproducibility of Radiomics in Magnetic Resonance Imaging: A Phantom Study. *Invest Radiol.* 2019;54:221-228.

**11.** Buckler AJ, Danagoulian J, Johnson K, et al. Inter-Method Performance Study of Tumor Volumetry Assessment on Computed Tomography Test-Retest Data. *Acad Radiol.* 2015;22:1393-1408.

**12.** Saltybaeva N, Tanadini-Lang S, Vuong D, et al. Robustness of radiomic features in magnetic resonance imaging for patients with glioblastoma: Multi-center study. *Phys Imaging Radiat Oncol.* 2022;22:131-136.

**13.** Burdick RK, Borror CM, Montgomery DC. Design and Analysis of Gauge R&R Studies: Making Decisions with Confidence Intervals in Random and Mixed ANOVA Models. *Design and Analysis of Gauge R&R Studies: Making Decisions with Confidence Intervals in Random and Mixed Anova Models.* 2005;17:1-201.

**14.** Woodall WH, Borror CM. Some relationships between gage R&R criteria. *Quality and Reliability Engineering International.* 2008;24:99-106.

**15.** Choe HM, Kim M, Lee EK. EMSaov: An R Package for the Analysis of Variance with the Expected Mean Squares and its Shiny Application. *R Journal.* 2017;9:252-261.

**16.** Bates D MM, Bolker B, Walker S lme4: Linear mixed-effects models using Eigen and S4. R package version 1.0-6. x. <http://CRAN.R-project.org/package=lme4>.

**17.** Parker RA, Scott C, Inacio V, Stevens NT. Using multiple agreement methods for continuous repeated measures data: a tutorial for practitioners. *Bmc Medical Research Methodology.* 2020;20:154.

**18.** Lin L, Hedayat AS, Sinha B, Yang M. Statistical methods in assessing agreement: Models, issues, and tools. *Journal of the American Statistical Association.* 2002;97:257-270.

**19.** Barnhart HX, Yow E, Crowley AL, et al. Choice of agreement indices for assessing and improving measurement reproducibility in a core laboratory setting. *Statistical Methods in Medical Research.* 2016;25:2939-2958.
