# Supplementary figures and images for: Reproducibility and repeatability of 18F-(2S, 4R)-4-fluoroglutamine PET imaging in preclinical oncology models
Source: PLoS One. 2025 Jan 9;20(1):e0313123. doi: 10.1371/journal.pone.0313123 (PMC11717184; doi:10.1371/journal.pone.0313123)

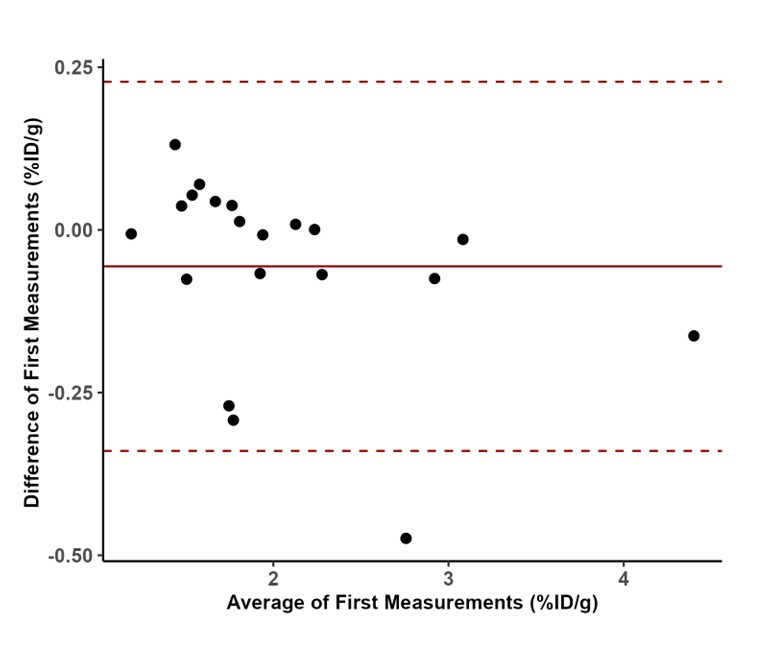

Supplement: S1 Fig — (TIF) [file pone.0313123.s002.tif]

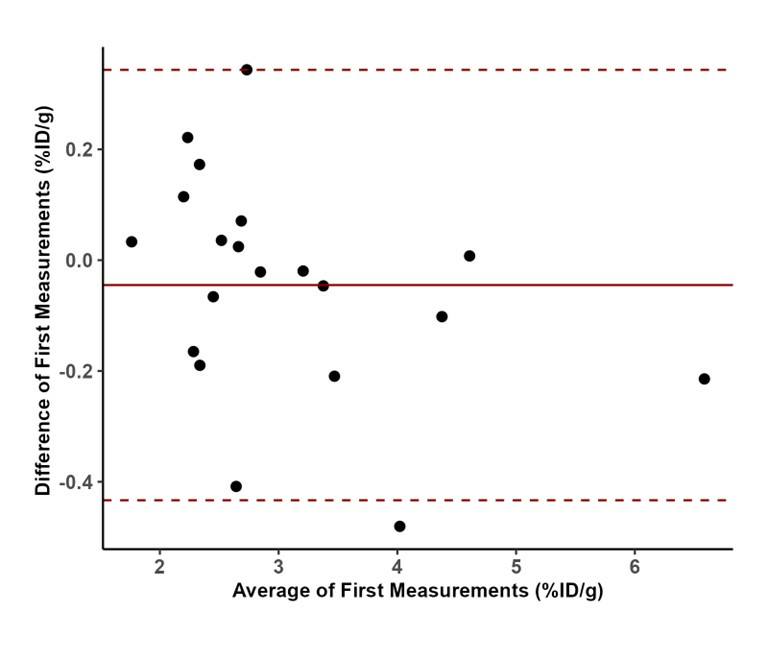

Supplement: S2 Fig — (TIF) [file pone.0313123.s003.tif]

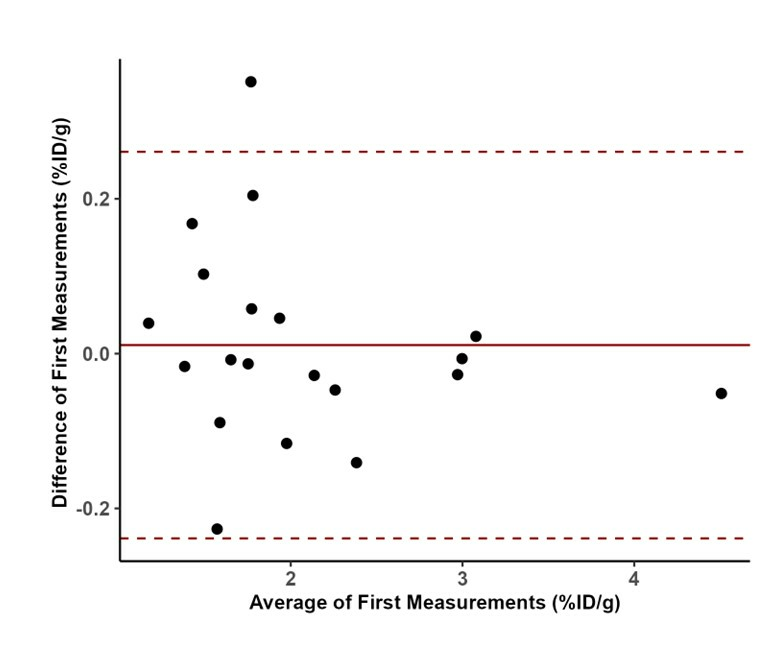

Supplement: S3 Fig — (TIF) [file pone.0313123.s004.tif]

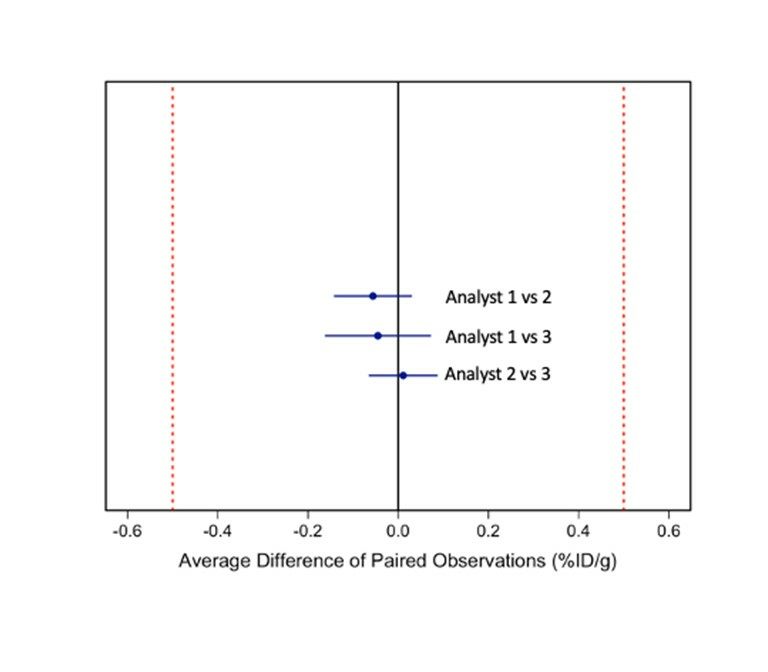

Supplement: S4 Fig — Shown are Bonferroni adjusted 98.3% (1–0.05/3) confidence intervals to control the experiment-wise type I error rate at 5%. (TIF) [file pone.0313123.s005.tif]

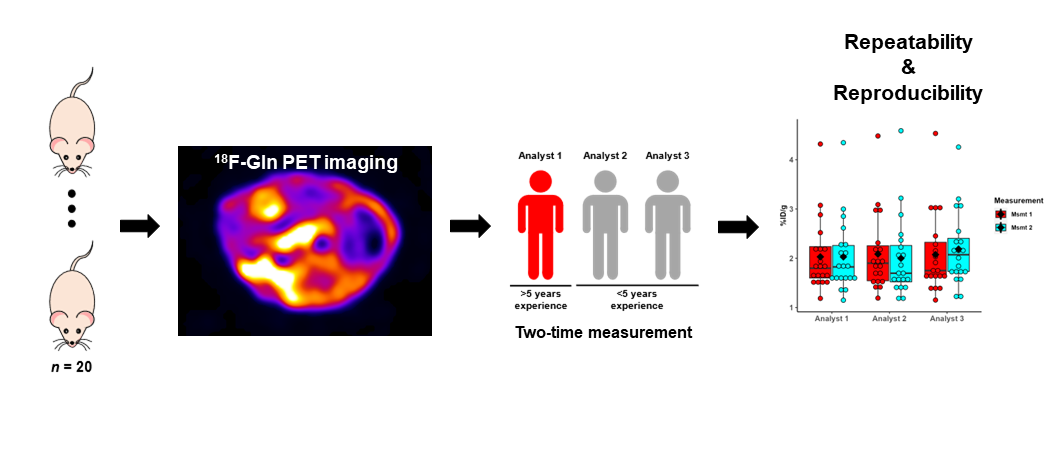

Supplement: S1 Graphical abstract — (TIF) [file pone.0313123.s010.tif]
