# Supplementary material for: Reproducibility and repeatability of 18F-(2S, 4R)-4-fluoroglutamine PET imaging in preclinical oncology models
Source: PLoS One. 2025 Jan 9;20(1):e0313123. doi: 10.1371/journal.pone.0313123 (PMC11717184; doi:10.1371/journal.pone.0313123)
Supplement: S1 Table — (DOCX) [file pone.0313123.s006.docx]

**S1 Table.** Summary statistics of Tumor/Muscle ratio by analyst and measurement.

| Statistic | Analyst 1 | | Analyst 2 | | Analyst 3 | |
| --- | --- | --- | --- | --- | --- | --- |
|  | Msmt1 | Msmt 2 | Msmt1 | Msmt 2 | Msmt1 | Msmt 2 |
| Mean (SD) | 1.09 (0.25) | 1.13 (0.22) | 1.11 (0.24) | 1.13 (0.26) | 1.17 (0.26) | 1.17 (0.25) |
| Median (range) | 1.04  (0.77, 1.53) | 1.07  (0.76, 1.57) | 1.15  (0.65, 1.47) | 1.03  (0.73, 1.65) | 1.14  (0.77, 1.64) | 1.21  (0.67, 1.66) |

*Msmt = measurement
