# Supplementary material for: Reproducibility and repeatability of 18F-(2S, 4R)-4-fluoroglutamine PET imaging in preclinical oncology models
Source: PLoS One. 2025 Jan 9;20(1):e0313123. doi: 10.1371/journal.pone.0313123 (PMC11717184; doi:10.1371/journal.pone.0313123)
Supplement: S2 Table — (DOCX) [file pone.0313123.s007.docx]

**S2 Table.** Results of gauge reproducibility and repeatability on first measurement data set.

| **Model Parameter** | **Analyst as a**  **Fixed Effect** | **Analyst as a**  **Random Effect** |
| --- | --- | --- |
| Mouse Variance | 0.580 | 0.580 |
| Analyst Variance | 0.0002 | 0.0002 |
| Error Variance | 0.013 | 0.013 |
| Mouse Variance | 0.580 (0.139, 1.123) | 0.580 (0.140, 1.134) |
| Measurement Variance | 0.013 (0.006, 0.021) | 0.013 (0.006, 0.022) |
| Mouse to Measurement Ratio | 44.992 (10.193, 128.110) | 44.712 (10.397, 128.103) |
| Repeatability Proportion | 0.987 (0.814, 1) | 0.981 (0.746, 1) |
| Reproducibility Proportion | 0.013 (0, 0.186) | 0.019 (0, 0.254) |
| Intraclass Correlation Coefficient | 0.978 (0.911, 0.992) | 0.978 (0.912, 0.992) |
