# Supplementary material for: Reproducibility and repeatability of 18F-(2S, 4R)-4-fluoroglutamine PET imaging in preclinical oncology models
Source: PLoS One. 2025 Jan 9;20(1):e0313123. doi: 10.1371/journal.pone.0313123 (PMC11717184; doi:10.1371/journal.pone.0313123)
Supplement: S3 Table — (DOCX) [file pone.0313123.s008.docx]

**S3 Table.** Bland-Altman Limits of Agreement (LOA) for first measurement.

| **Analysts** | **1/2 Agreement Limit^*^** | **Equivalency Lower Confidence Limit^**^** | **Bias^***^** | **Equivalency Upper Confidence Limit^**^** |
| --- | --- | --- | --- | --- |
| 1 vs 2 | 0.284 | -0.124 | -0.056 | -0.056 |
| 1 vs 3 | 0.388 | -0.138 | -0.045 | -0.045 |
| 2 vs 3 | 0.250 | -0.049 | 0.011 | 0.071 |

^*^LOA = 1.96*S_d_, where S_d_ is the standard deviation of paired differences. No repeatability index when repeats not made. ^**^95% confidence intervals for the average difference using t value with 19 degrees of freedom. ^***^Average difference of paired measurements.
