# Supplementary material for: Reproducibility and repeatability of 18F-(2S, 4R)-4-fluoroglutamine PET imaging in preclinical oncology models
Source: PLoS One. 2025 Jan 9;20(1):e0313123. doi: 10.1371/journal.pone.0313123 (PMC11717184; doi:10.1371/journal.pone.0313123)
Supplement: S4 Table — (DOCX) [file pone.0313123.s009.docx]

**S4 Table.** Concordance Correlation Coefficient (95% confidence interval) for first measurement between analysts.

| **Analysts** | **Offset from Agreement Line (Cb)** | **Pearson’s Correlation**  **(r=precision)** | **Concordance Correlation Coefficient ^*^** |
| --- | --- | --- | --- |
| 1 vs 2 | 0.995 | 0.984 | 0.98 (0.95 to 0.99) |
| 1 vs 3 | 0.993 | 0.972 | 0.96 (0.92 to 0.98) |
| 2 vs 3 | 0.999 | 0.988 | 0.99 (0.97 to 0.99) |

^*^ CCC = Cb*r
